# Safety of sequential immune checkpoint inhibitors after prior immune therapy

**DOI:** 10.1007/s00432-022-04137-4

**Published:** 2022-06-21

**Authors:** Muhammad Awidi, Brendan Connell, Delaney Johnson, Isabel Craven, Rojer Ranjit, Brigitte Gil, Natalie Dal’Bo, Lewena Maher, Seanna Reilly Daves, Stephanie McDonald, Krishna S. Gunturu

**Affiliations:** 1 Internal Medicine Department, Lahey Hospital and Medical Center, Burlington, MA USA; 2 Department of Oncology, Lahey Hospital and Medical Center, Burlington, MA USA; 3 Tufts University School of Medicine, Boston, MA USA; 4 Boston College, Chestnut Hill, MA USA; 5 Department of Oncology, Massachusetts General Hospital, Boston, MA USA; 6 Department of Pharmacy, Lahey Hospital and Medical Center, Burlington, MA USA; 7 Department of Rheumatology, Roger Williams Medical Center, Rhode Island, USA; 8 Department of Cardiology, Massachusetts General Hospital, Boston, MA USA

**Keywords:** Immune checkpoint inhibitors, Rechallenge, Sequential, Immune related adverse events, Retreatment, Reintroduction

## Abstract

**Background:**

The use of immune checkpoint inhibitors (ICI) has transformed cancer treatment. Subsequent ICI use has become increasingly common following disease progression. We aim to evaluate the safety and tolerability of the sequential ICI treatment modality.

**Methods:**

Retrospective review of confirmed carcinoma from January 2014 to December 2018. Patients were categorized into “initial ICI arm” and “sequential ICI arm” defined as patients receiving single, dual or chemo-immunotherapy ICI following an initial ICI regimen. Primary outcome was the development of a new or recurrent immune related adverse event (irAE) during sequential therapy. Secondary outcomes were the number of cycles prior to the development of irAE and grade of irAE.

**Results:**

A total of 483 patients received ICI during the timeframe. Of those, 22 patients received sequential ICI. The diagnoses included ten lung cancer, seven melanoma, four renal cell carcinoma and one bladder cancer. 16 patients received single agent ICI following the initial ICI, three patients received dual ICI following the initial ICI, one patient received chemotherapy-immunotherapy following initial ICI, and two patients received chemo-immunotherapy after dual ICI. Four patients developed new irAE and one patient developed the same irAE on sequential treatment. A higher proportion of patients experienced grade 3 irAE in the sequential arm compared to the initial ICI arm (*p* = 0.03). No statistical difference was found between the development of irAE and the number of cycles prior to development of irAE in either treatment groups (*p* = 0.5).

**Conclusion:**

Our data shows overall safety of sequencing ICI when close monitoring was employed.

## Introduction

The use of immune checkpoint inhibitors (ICI) has transformed cancer treatment and has become a regular treatment option for many oncologists. ICI’s stimulate the immune system’s antitumor response and suppress the cytotoxic T-lymphocyte antigen-4 (CTLA-4), programmed cell death 1, or ligand 1 (PD-1/PDL-1) (Larkin et al. 2019). Presently, ICI treatment is FDA approved to treat many types and stages of malignancies including colorectal cancer, pleural mesothelioma, breast cancer, bladder cancer, endometrial carcinoma, esophageal squamous cell carcinoma, lung cancer, renal cell carcinoma, Merkel cell carcinoma, hepatocellular carcinoma, lymphoma, cervical cancer, gastric cancer, head and neck cancer, and urothelial carcinoma (Twomey and Zhang 2021). ICI utilization has greatly increased due to the clinical benefits and significant improvements in progression free survival and overall survival (Postow et al. 2015). Different treatment strategies with sequential and combination ICI are being investigated and have shown to be effective with a higher immunogenic effect compared to single agent ICI in certain cancers (Winer et al. 2019; Curran et al. 2010).

The significant benefits of ICI must be weighed against their toxicities, most notably immune related adverse events (irAEs) (Gunturu et al. 2022). Any organ can be affected by irAEs and they occur with varying degrees of severity. Generally, treatment with ICI can continue with grade 1 irAEs with close monitoring. Grade 2 irAEs may warrant temporary discontinuation of ICI until toxicity reverts to grade 1 or less, and irAEs above grade 2 generally warrant suspension or permanent discontinuation of ICI therapy (Brahmer et al. 2018) in addition to corticosteroids or other immune suppressing medications. Data on the safety and efficacy of ICI in patients who previously develop irAEs has been extensively reported (Zhao et al. 2021). Following disease progression on ICI, the use of a subsequent ICI has become increasingly common for patients with severe disease, but the safety and efficacy of sequential therapy have not been well studied.

We conducted a retrospective review of patients who received a second ICI treatment either as a monotherapy or in combination with another ICI or chemotherapy and evaluated the safety and tolerability of sequential treatment modality.

## Methods

This was a retrospective study that included all patients with a histology-confirmed carcinoma who received subsequent ICI following initial ICI therapy. ICI regimens included anti-CTLA-4 (ipilimumab), anti-PD1/PDL1 (nivolumab, pembrolizumab, durvalumab, atezolizumab), dual anti-CTLA-4 and anti-PD-L1 (ipilimumab and nivolumab) or chemotherapy-ICI combination between January 1, 2014 and December 18, 2018. We collected baseline patients and tumor characteristics, details of the ICI regimen used and irAEs data. Prior to initiating the study, approval was obtained from the Beth Israel Lahey Health Institutional Review Board.

We defined and graded the severity of irAEs according to the Common Terminology Criteria for Adverse Events Version 4.0. We categorized patients into “Initial ICI” arm and “Sequential ICI” arm defined as patients receiving a second ICI either as a single, dual ICI or a chemotherapy-ICI combination. Common side effects included skin toxicities, gastrointestinal (GI) toxicity, pneumonitis and endocrinopathy. Skin toxicity was defined to include maculopapular rash, dermatitis, and bullous pemphigoid. GI toxicity included colitis and hepatitis. Endocrinopathy included thyroiditis and adrenal insufficiency.

The primary outcome was the development of a new or recurrent irAEs during sequential therapy. Secondary outcomes were number of cycles prior to the development of irAEs and grade of irAEs in patients receiving sequential therapy.

### Statistical analysis

Statistical analysis was performed using R statistical software (v4.0.3). Descriptive and univariate analyses were conducted to provide an overview of study population characteristics. We analyzed the primary and secondary outcomes via Fisher’s exact test and Wilcoxon rank sum test when appropriate. We set *p* value of < 0.05 to define statistically significant outcomes.

## Results

A total of 483 patients received ICI in the time frame. Of those, 22 patients received sequential ICI as shown in Table 1. 16 patients (72%) received single agent ICI following the initial ICI, three patients (14%) received dual ICI following the initial ICI, one patient (5%) received chemotherapy-ICI following initial ICI, and two patients (9%) received chemo-ICI after dual ICI.

**Table 1:** General characteristics

Characteristics	Initial treatment (*n* = 22)	Sequential treatment (*n* = 22)	*P* value
Type of ICI			≤ 0.01
Anti-CTLA-4			
Ipilimumab	6 (27%)	0 (0%)	
Anti-PD-1			
Nivolumab	12 (55%)	6 (27%)	
Pembrolizumab	0 (0%)	10 (45%)	
Anti-PDL-1			
Durvalumab	1 (4%)	0 (0%)	
Atezolizumab	1 (4%)	0 (0%)	
Anti-CTLA-4 and anti-PD-1 combination			
Nivolumab and ipilimumab	2 (9%)	3 (14%)	
Anti-PD-1 and chemotherapy combination			
Pembrolizumab, Carboplatin, Pemetrexed	0 (0%)	3 (14%)	
IrAE events, n (%)			
	10 (45%)	7 (31%)	0.5
Type of irAE, n (%)			
Adrenalitis	0 (0%)	1 (14%)	0.2
Bullous pemphigoid	0 (0%)	1 (14%)	
Colitis	1 (10%)	0 (0%)	
Conjunctivitis	1 (10%)	0 (0%)	
Dermatitis	6 (60%)	2 (29%)	
Hypothyroidism	2 (20%)	1 (14%)	
Pneumonitis	0 (0%)	2 (29%)	
Grade of irAE, n (%)			0.03
1	5 (50%)	2 (29%)	
2	4 (40%)	0 (0%)	
3	1 (10%)	5 (71%)	
ICI dose prior to irAE, n (%)			0.5
1	0 (0%)	1 (14%)	
2	2 (20%)	2 (29%)	
3	2 (20%)	1 (14%)	
4	4 (40%)	1 (14%)	
5	0 (0%)	1 (14%)	
11	1 (10%)	0 (0%)	
12	0 (0%)	1 (14%)	
15	1 (10%)	0 (0%)	

All patient except one received sequential ICI due to disease progression. Patient 12 received sequential nivolumab after ipilimumab following the development of severe colitis.

Of the 22 patients, the diagnoses included lung cancer (*n* = 10), melanoma (*n* = 7), renal cell carcinoma (*n* = 4) and bladder cancer (*n* = 1). Histological subtypes of lung cancer were adenocarcinoma (*n* = 7) and squamous cell carcinoma (*n* = 3). Histological subtypes in renal cell carcinoma were clear cell (*n* = 3) and papillary (*n* = 1) and urothelial carcinoma in bladder cancer (*n* = 1).

The most common initial ICI used was nivolumab (*n* = 12, 52%) followed by ipilimumab (*n* = 7, 30%), Combined nivolumab and ipilimumab (*n* = 2, 8.7%), atezolizumab (*n* = 1, 4.3%), and durvalumab (*n* = 1, 4.3%). None of the patients received pembrolizumab as an initial ICI. However, pembrolizumab accounted for 59% (*n* = 13) followed by nivolumab for 41% (*n* = 9) of sequential ICI. Table 2 summarizes the diagnostic, therapeutic and irAEs details.

**Table 2:** Course of ICI therapy and irAEs

Patient number	Cancer type	Histo-pathology	Initial treatment of ICI					Sequential treatment of ICI				
			Type of ICI*	irAE			Intervention	Type of ICI*	irAE			Intervention
				Type	Grade	# ICI doses			Type	Grade	# ICI doses	
1	Bladder	Urothelial	Atezo	–	–	–	–	Pembro	–	–	–	–
2	Lung	Squamous	Nivo	–	–	–	–	Pembro	–	–	–	–
3	Melanoma	Melanoma	Ipili	Dermatitis	1	3	Betamethasone ointment until resolution	Nivo	Adrenalitis	3	3	Prednisone, hydrocortisone, fludrocortisone until resolution. Hospitalization required
4	Melanoma	Melanoma	Ipili	–	–	–	–	Pembro	Dermatitis	1	1	Hydrocortisone until resolution
5	Melanoma	Melanoma	Ipili	Dermatitis	2	4	Triamcinolone until resolution	Nivo	–	–	–	–
6	Lung	Adeno-carcinoma	Nivo	–	–	–	–	Pembro	–	–	–	–
7	Lung	Adeno-carcinoma	Nivo	–	–	–	–	Pembro	Pneumonitis	3	12	Prednisone until resolution. Hospitalization required
8	Melanoma	Melanoma	Ipili	Dermatitis	1	4	Betamethasone ointment until resolution	Nivo	–	–	–	–
9	Melanoma	Melanoma	Ipili	Dermatitis	1	4	Betamethasone ointment until resolution	Nivo	–	–	–	–
10	Lung	Adeno-carcinoma	Nivo	–	–	–	–	Pembro	Dermatitis	1	5	Betamethasone ointment until resolution
11	Lung	Squamous	Nivo	–	–	–	–	Pembro	–	–	–	–
12	Melanoma	Melanoma	Ipili	Dermatitis Colitis	3 2	2	Prednisone until resolution	Nivo	Pneumonitis	3	2	Prednisone until resolution. Hospitalization required
13	Renal	Clear Cell	Nivo	–	–	–	–	Pembro	–	–	–	–
14	Lung	Adeno-carcinoma	Nivo	–	–	–	–	Pembro	–	–	–	–
15	Lung	Adeno-carcinoma	Nivo	Dermatitis	1	15	Betamethasone until resolution	Pembro	Lost to follow up			
16	Renal	Clear Cell	Nivo	–	–	–	–	Nivo	Bullous Pemphigoid	3	4	Prednisone and methotrexate until resolution
17	Renal	Papillary	Nivo	Hypothyroidism	2	3	Levothyroxine ongoing	Nivo&Ipili	Hypothyroidism	3	2	Levothyroxine ongoing
18	Melanoma	Melanoma	Nivo	Conjunctivitis	1	11	Olopatadine eye drops until resolution	Nivo&Ipili	–	–	–	–
19	Renal	Clear Cell	Nivo	–	–	–	–	Nivo&Ipili	–	–	–	–
20	Lung	Adeno-carcinoma	Durva	–	–	–	–	Pembro,Carbo/Peme	–	–	–	–
21	Lung	Adeno-carcinoma	Nivo, Ipili	Hypothyroidism	2	4	Levothyroxine ongoing. Hospitalization required	Pembro,Carbo/Peme	–	–	–	–
22	Lung	Squamous	Nivo, Ipili	–	–	–	–	Pembro,Carbp/Peme	–	–	–	–

^*^ICI abbreviations listed in the table are as follows: pembrolizumab (Pembro), nivolumab (Nivo), durvalumab (Durva), ipilimumab (Ipili), Carboplatin (Carbo), Pemetrexed (Peme)

Overall, 9 patients (41%) had irAEs after the initial exposure to ICI resulting in 10 irAEs. Initial irAEs included skin toxicity (*n* = 6, 60%), endocrinopathy (*n* = 2, 20%), colitis (*n* = 1, 10%), and conjunctivitis (*n* = 1, 10%). In patients receiving initial ICI five (50%) had grade 1 irAEs, four (40%) had grade 2 irAE and one (10%) had grade 3 irAEs. Table 3 summarizes the details of irAEs.

**Table 3:** Frequency of irAEs

Immune-related adverse events (irAEs)	Initial treatment		Sequential treatment	
	Any grade	Grade 3+	Any grade	Grade 3+
Dermatitis	6	1	2	0
Colitis	1	0	0	0
Hypothyroidism	2	0	1	1
Pneumonitis	0	0	2	2
Conjunctivitis	1	0	0	0
Adrenalitis	0	0	1	1
Bullous pemphigoid	0	0	1	1
Total	10 irAE across 9/22 patients	1/10 (10%) irAEs were Grade 3+	7 irAE across 7/22 patients	5/7 (71%) irAEs were Grade 3+

In patients receiving sequential treatment, seven of the 22 developed irAEs. Of these seven, skin toxicity accounted for 43% (*n* = 3), pneumonitis for 29% (*n* = 2), and endocrinopathy for 29% (*n* = 2). As for grading, two patients (29%) in the sequential group had grade 1 irAEs, five patients (71%) had grade 3 irAE and none developed grade 2 irAEs. One patient with melanoma developed grade 1 dermatitis with initial ipilimumab use and then developed grade 3 adrenalitis on sequential nivolumab use. Another patient with papillary renal cell carcinoma had a higher grade (3 vs 2) of the same irAEs (hypothyroidism) with the sequential use of nivolumab. No statistical difference was found between the development of irAEs and the number of cycles prior to development of irAEs in either treatment groups (*p* = 0.5).

Interestingly, a higher proportion of patients experienced grade 3 irAEs in the sequential ICI group (*n* = 5, 71%) compared to the initial ICI group (*n* = 1, 10%) (*p* = 0.03). As shown in Table 4. Notably one patient with hypothyroidism developed the same but a higher grade irAE, two patients developed new grade 3 irAEs only with sequential treatment (pneumonitis, bullous pemphigoid) with the other two patients developing a different higher grade irAEs on sequential therapy as shown in Table 2.

**Table 4:** Grade of irAE

irAE grade*	Initial treatment	Sequential treatment
Grade 1	5	2
Grade 2	4	0
Grade 3	1	5

*p value = 0.03

## Discussion

ICI revolutionized the treatment and prognosis of many cancer patients. Despite major advances, developing treatment strategies to maximize efficacy of ICI treatments while balancing toxicity profiles remain one of the biggest challenges faced by medical oncologists today. The incidence of grade 3 and 4 irAEs has been reported to be 14% after anti-PD-1 monotherapy (Topalian et al. 2012), 23% after anti-CTLA-4 monotherapy (Boutros et al. 2016) and 53% after combination therapy (Wolchok et al. 2013).

Studies exploring the safety of ICI rechallenge after the development of irAEs are not conclusive with some studies demonstrating ICI rechallenge is safe and efficacious by comparing the incidence of irAEs with initial and secondary rechallenge of ICI (Abu-Sbeih et al. 2019; Ravi et al. 2020). A recent metanalysis showed that patients rechallenged with ICI had higher incidence for all-grade irAEs (OR 3.81, *p* < 0.0001), but similar incidence for high grade irAEs (*p* > 0.05) (Zhao et al. 2021). However, the utility and safety of sequential immunotherapy is not yet known, and studies are still lacking.

Preclinical data has shown that some anti-PD-1/PDL-1 bind to different epitopes (Martini et al. 2017). This has been used as the basis to use them as sequential therapy with data suggesting the benefit of the addition of anti-CTLA-4 to anti-PD-1 upon progression of disease on a single ICI (Gide et al. 2018; Babacan and Eroglu 2020; Robert et al. 2014). We report the safety and rate of irAEs of 22 patients receiving sequential ICI.

In our study, sequential ICI therapy was not associated with a higher rate of irAEs but was associated with an increase in irAEs grade if patient had incurred an irAEs.

In our study, all but one of our patients received sequential ICI due to disease progression with one being rechallenged with a different ICI after the development of irAEs.

Most patients in the initial treatment arm developed irAEs after receiving an average of 6 cycles with two patient developing conjunctivitis and dermatitis after 11 and 15 cycles respectively. In the sequential treatment group, irAEs developed after an average of 4 cycles. One patient was lost to follow-up thus possible influencing the analysis. The time to develop irAE was consistent with what is currently reported in the literature (Tang et al. 2021). Nonetheless, there were no statistically significant difference between the number of cycles and the grade of irAEs.

Recent studies show that the rate of irAEs in melanoma patients was about 49% in patients treated with anti-PD1 (Indini et al. 2019). In our melanoma cohort, irAEs rate in initial treatment arms were 85% (*n* = 6) and 43% (*n* = 3) in the sequential arm. The difference in irAEs between the two arms could be due to the small number of patients in the initial ICI group, as well as the different ICI used, which is known to have a different irAEs incidence (Weber et al. 2017). In a study of patients with metastatic melanoma who progressed on first line anti-PD-1 therapy treated with ipilimumab and an anti-PD-1, five out of 15 (33%) patients developed grade 3–4 irAEs leading to treatment discontinuation (Mehmi and Hill 2018). In another study in patients with advanced melanoma who had progressed on anti-PDL-1/L1 antibodies received combination pembrolizumab plus ipilimumab followed by Pembrolizumab monotherapy, treatment related adverse events occurred in 62 of 70 patients with grade 3–4 occurring in 19 of 70 patients (27%) (Olson et al. 2021).

In a retrospective study of metastatic renal cell carcinoma, Ravi et al reported a 16% grade ≥ 3 irAEs after rechallenge due to disease progression (72%) or intolerable ICI side effects (23%). (Ravi et al. 2020). Gul et al. reported 45 patients receiving salvage ipilimumab plus nivolumab after anti-PDL-1 therapy with grade 3 irAEs reported in six patients (13%) (Gul et al. 2020). However, in the OMNIVORE trial, patients with advanced renal cell carcinoma with stable disease or disease progression after nivolumab received two doses of ipilimumab. Any grade and grade ≥ 3 treatment related adverse event occurred in 81% and 25% of patients respectively (McKay et al. 2020). In our patients with RCC, incidence of irAEs was 25% in the initial and 50% in the sequential groups respectively.

In a study of bladder urothelial carcinoma patients who received ipilimumab and nivolumab at disease progression reported a grade ≥ 3 irAE of 43% (Keegan et al. 2019). We reported one patient with bladder urothelial carcinoma who received pembrolizumab after atezolizumab without any irAEs.

In our patients with non-small cell lung cancer, irAEs rate in both arms were 20% (*n* = 2). IrAEs in the initial treatment group were grade 1. In the sequential group, however, one of the patients developed a grade 3 irAE. In a retrospective study, evaluating the safety and efficacy of pembrolizumab after nivolumab. 12 patients with NSCLC were analyzed. The nivolumab group had grade 1 and grade ≥ 2 irAE of seven and eight respectively. While the pembrolizumab group had grade 1 and grade ≥ 2 irAE of 6 and 10 respectively (Fujita et al. 2018).

Given the scarcity of prospective data on ICI rechallenge, there are currently no available evidence-based guidelines on how to safely approach this therapeutic modality. The limited studies are available have suggested methods to minimize recurrence of irAEs in patients rechallenged with ICI.

Studies suggest that a longer delay between ICI discontinuation due to irAE and rechallenge was associated with lower rate of recurrence of irAEs. Furthermore, based on studies that draw attention to the fact that premature discontinuation of ICI due to severe irAEs was not associated with worse outcomes (Schadendorf et al. 2017; Horiguchi et al. 2018). Delyon et al. (2019) waited for disease progression before ICI rechallenge with less reported irAEs in patients with melanoma. However, this has not been confirmed in nonmelanoma tumors. Some lung cancer studies suggest resuming ICI after irAE improved outcomes in patients who had not yet achieved response (Santini et al. 2018). In addition, Allouchery et al. showed that the rechallenge with the same ICI drug or the same ICI combination was associated with a lower rate or irAE occurrence (Allouchery et al. 2020).

The decision to rechallenge patients with irAE presents a unique challenge for Oncologists. Until further evidence emerges a multidisciplinary approach should be taken to balance the risk and benefits of rechallenge.

Finally, our study has several limitations. Given the retrospective nature of the study and the small sample size included, our ability to draw definitive conclusions is limited. However, we report findings in an understudied but increasingly encountered scenario in the clinical practice where there is no clear data is available. Further prospective studies are required to validate the findings and establishing treatment guidelines in patients receiving sequential immune checkpoint inhibitor therapy.

## Conclusion

Currently, there is insufficient data on the safety and efficacy on the sequential use of ICI after prior ICI. Our data shows overall safety of sequencing ICI when close monitoring was employed. Ideally, prospective studies to investigate the safety and efficacy of sequential therapy are needed.

## Data Availability

The datasets generated during and/or analyzed during the current study are available from the corresponding author on reasonable request.
